# Neophobia across social contexts in juvenile herring gulls

**DOI:** 10.1098/rsos.250398

**Published:** 2025-05-21

**Authors:** Reinoud Allaert, Sophia Knoch, Simon Braem, Dries Debeer, An Martel, Wendt Müller, Eric Stienen, Luc Lens, Frederick Verbruggen

**Affiliations:** ^1^ Department of Biology, Ghent University, Ghent, Belgium; ^2^ Centre for Research on Ecology, Cognition and Behaviour of Birds, Ghent University, Ghent, Belgium; ^3^ Department of Experimental Psychology, Ghent University, Ghent, Belgium; ^4^ Research Support Office, Faculty of Psychology and Educational Sciences, Ghent University, Ghent, Belgium; ^5^ Deptartment of Pathobiology, Pharmacology and Zoological Medicine, Ghent University, Merelbeke, Belgium; ^6^ Department of Biology, Antwerp University, Antwerp, Belgium; ^7^ Research Institute for Nature and Forest, Brussels, Belgium

**Keywords:** animal behaviour, behavioural inhibition, neophobia, social behaviour, herring gull, animal personality

## Abstract

Neophobia, the fear or avoidance of the unfamiliar, can have significant fitness consequences. It is typically assessed by exposing individuals to unfamiliar objects when they are alone, but in social species, the presence of conspecifics can influence neophobia. However, previous research on the effect of group dynamics on neophobic responses has produced mixed results. Here, we explored the degree of neophobia of an individual in different social contexts in a highly social species, the herring gull. To this end, we exposed juvenile herring gulls (*n* = 54) to novel objects in both individual and group settings (4–5 individuals), replicating each condition twice. Individuals tested in groups were quicker to eat and spent more time near a novel object than individuals tested alone. The results of our study suggest that the presence of group members reduces perceived individual risk, allowing individuals to behave less cautiously. Preregistered Stage 1 protocol: https://osf.io/u4b7q (date of in-principle acceptance: 17 May 2024).

## 1. Introduction

Neophobia is the fear or reluctance to engage with new or unfamiliar objects, places or scenarios. It is often considered to be a consistent personality trait across species, affecting an individual’s survival and adaptation [1–4] . Research into animal behaviour is increasingly focusing on neophobia because of its significance in the context of rapid environmental change. The world is rapidly urbanizing, with the footprint of urban land cover expected to at least double by the end of the century [5]. Many species must, therefore, adapt to human-induced changes in their environment, and hence, to unfamiliar scenarios [6,7]. In such situations, neophobia can, on the one hand, serve as a survival mechanism, allowing individuals to avoid potential threats and increase their chance of survival [8]. On the other hand, excessive aversion to novelty can restrict exploratory behaviour, limiting an individual’s ability to locate and exploit novel resources, learn from its novel environment and adapt to environmental changes [9,10].

To assess neophobia, individuals are typically exposed to novel food, objects or spaces [2,10]. For example, in the ‘novel object task’, an individual encounters an unfamiliar object, often placed next to a food reward, in a familiar environment. The latency to approach the food (in the presence of the novel object) or to interact with the novel object itself, is then used as a measure of neophobia [2,12,13]. These measures have been used in cross-species comparisons to investigate, for example, the socio-ecological drivers of neophobia [12,14,15 ], or within species, to investigate both the causes and consequences of individual differences in neophobia [8].

Most research on neophobia has focused on individual animals, both in laboratory and field settings. However, it is important to consider that many species are, to various extents, reliant on social information, so individuals can influence each other’s behaviour. This is also true in the context of adapting to environmental changes and urbanization [6]. For instance, when individuals encounter a new environment, they may learn from others about appropriate roosting or nesting sites, food sources, or unfamiliar predators [16–18]. In this context, several studies suggest that the presence of conspecifics also influences neophobia. However, the mechanisms behind this social phenomenon are still a topic of debate due to the various patterns that have been observed.

First, some studies have found that individuals in groups are generally less neophobic than when tested alone. For example, Coleman and Mellgren presented zebra finches (*Taeniopygia guttata*) with novel feeders and decorated the feeders with novel objects [19]. Individuals in a group approached and started using the new and decorated feeders more quickly than when tested alone. Other studies reported similar patterns in different species for some (but not necessarily all) measures of neophobia [20–23]. Such mitigating effects of social context on neophobia may be attributed to ‘risk dilution’ [24] or ‘social buffering’ [25]. These theories predict that neophobia, or fear responses in general, are reduced in the presence of others, as individuals in a group collectively share the potential risks associated with novel situations or threats. This shared risk perception will also lead to more uniform behaviour within the group, as individuals adapt their actions in response to the behaviour of conspecifics.

Second, some studies found the opposite pattern. For example, common ravens (*Corvus corax*) and carrion x hooded crows (hybrid; *C. corone, C. cornix*) approached novel objects faster when alone than when accompanied by a conspecific [26,27]. Other studies have observed similar patterns in other species, including Indian mynahs, *Acridotheres tristis* [28], house sparrows, *Passer domesticus* [29] and even zebra finches [30,31], thus failing to replicate the findings of the aforementioned study by Coleman and Mellgren [19]. Interestingly, however, some of these studies found that once individuals reached the novel object, they spent more time interacting with it when in the presence of others (either in pairs or in groups) than when isolated [26,31]. It has therefore been suggested that the slower approach latencies may be due to conspecifics ‘negotiating’, by using behavioural cues to coordinate their actions and deciding who will approach the novel object first. Consequently, this may lead to a convergence of individual behaviours, as group members align their actions based on these cues.

Third, some studies failed to find the effects of social context on average neophobic responses altogether (e.g. [32]). While it is, of course, possible that social context does not matter for some species, it is also possible that the presence of conspecifics alters the behaviour of individuals without changing the mean response. Specifically, in environments where conspecifics’ behaviour serves as an indicator of appropriate responses, individuals may adjust their own behaviour to match that of others [33]. This synchronization of behaviours within the group, or ‘social conformity’, enhances cohesion and helps the group to adapt to their environment. Observations in a variety of species, such as zebra finches [34] and Gouldian finches, *Erythrura gouldiae* [35], show how individuals adapt their behaviour and mirror their partners’ character traits. For instance, if a gouldian finch exhibited bold behaviour, the observing individual tended to become bolder as well, while if the partner displayed shyness, the observing individual mirrored this trait [35]. Thus, this study found that the neophobic response was similar on average for individuals tested alone or in pairs, but there was less variation between individuals in the paired condition compared to the alone condition.

**Current study:** The aim of this study is to investigate if and how the social context affects neophobia in the herring gull (*Larus argentatus*). Gulls’ natural coastal habitat is rapidly disappearing, forcing them to live closer to humans in urban environments and to rely more on anthropogenic food sources [36,37]. Although reports in popular media may suggest that herring gulls are generally not neophobic due to their approach towards humans or stealing food, such anecdotes do not necessarily reflect the species’ behaviour at a population level [38]. In fact, widely differing levels of neophobia as well as individual differences therein exist within populations [38]. The latter finding suggests that for some individuals, it might be easier to adapt to environmental change and urbanization than for others. Indeed, there is considerable intraspecific variation in how herring gulls utilize urbanized areas, ranging from minimally to almost complete dependence [39,40].

**Figure 1.**
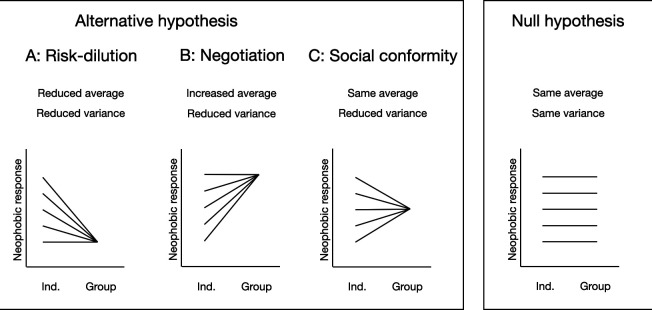
Overview of hypotheses.

Herring gulls are a highly social species, utilizing cues not only from conspecifics, but even from other species, including humans. This suggests that social learning is a key aspect of gull behaviour [41–44]. Thus, when assessing their neophobia, it is important to do this not only in an individual context, but also in a social (group) context. Based on previous findings (as reviewed above), we predict that the distribution of neophobic responses will depend on the social context. However, the direction of the effects will depend on the social mechanisms at play. In figure 1, we provide a template for testing the three different hypotheses of group effects, taking into account two measures, namely the average neophobic response and the variance between individuals.

Overall, we predict that there will be lower variance between individuals when they are tested in a group, compared to when they are tested alone. After all, all of the major hypotheses discussed above assume that individuals become more similar to each other by spreading risk, jointly buffering stress, negotiating with each other or simply through social conformity. However, there are three possible scenarios regarding the average neophobic response. First, the ‘risk dilution’ hypothesis predicts that herring gulls will be *less* neophobic on average when in a group compared to when they are alone (scenario A in figure 1). Second, the ‘negotiation’ hypothesis predicts that individuals will be *more* neophobic when in group (scenario B in figure 1). Third, according to the ‘social conformity’ hypothesis, individuals will tend to mimic one another’s behaviours—those who are neophobic will show a decrease in their fear of novel objects when surrounded by others who are less neophobic, and vice versa (scenario C in figure 1). Thus, in this third scenario, there is a reduction of variance but no change in the average neophobic response. These three predictions are contrasted with the null hypothesis that social context does not modulate variance, or group means (‘Null Hypothesis’, figure 1).

To test these predictions, juvenile herring gulls were subjected to four distinct conditions: individual or group tests paired with a control or novel object. Each condition was repeated twice. The guidelines for designing neophobia tests of [2] were followed, and a within-subject design with a relatively large sample size (*n* = 67 individuals) was chosen to further increase the statistical power of the study. One additional reason for the inconsistent previous findings is that sample size was relatively low in many studies (see also [45]). In addition, the herring gulls used in this study were raised by hand from the egg to control for sampling bias, a recurring issue when testing wild animals. After testing, they were released in the wild.

## 2. Material and methods

### 2.1. Sample size

We originally planned to test 80 herring gulls twice across a 2×2 design (thus eight tests per individual; see above). We performed an *a-priori* power sensitivity analysis using G*Power [46] for a repeated measures MANOVA with three within-subject factors: *context* (with levels group and individual), *object* (with levels control and novel object) and *trial* (with levels 1 and 2). Our initial analysis indicated that a sample size of 80 would be sufficient to detect small main effects of *context, object and trial* (Cohen’s f effect size of 0.11 [47]; Power = 0.80; correlation among repeated measures = 0.5), as well as an interaction between context and object with small effect size (0.11; Power = 0.80; correlation among repeated measures = 0.5). We reared the gulls from the eggs (see the §2.2 below) and we anticipated that in some cases herring gull eggs would be mistaken for those of the phylogenetically and ecologically related lesser black-backed gull (LBBG) during egg collection. For logistical reasons, the chicks could only be identified at the species level after testing by visual inspection of plumage differences. To mitigate the potential reduction in sample size (due to the exclusion of LBBGs), we conducted a second *a-priori* power analysis accounting for a potential 10% dropout rate. This *a-priori* analysis revealed that even with a 10% reduction, our study would still have sufficient statistical power (Cohen’s f effect size of 0.17) to detect significant effects.

Due to unanticipated mortality, we were only able to test 67 birds (instead of the registered 80). Of these 67 birds, 13 individuals were later identified as LBBG (a higher percentage than we had anticipated) and were excluded from further analysis in accordance with the registered protocol as there may be differences in neophobic responses between migratory (i.e. LBBG) and non-migratory (i.e. herring gulls) species [15]. This further reduced our final sample size (*n* = 54). Although this is a significant reduction from our planned sample size (*n* = 72 after exclusion of LBBG), it is important to note that our sensitivity analyses were based on repeated measures MANOVAs (within-subjects factors). This type of analysis does not take into account the additional flexibility offered by (G)LMMs, which are not currently covered by G*Power or most other power estimation tools. The mixed effects models used in this study (in line with the registered protocol) are more robust and better equipped to deal with unexplained variance than the fixed effects MANOVAs used in our sensitivity analysis. Thus, despite the reduction in sample size, our proposed mixed-effects models are expected to retain sufficient power to detect the effects of interest. An overview of the group composition, including the number of LBBG individuals and the sex distribution, is provided in electronic supplementary material, table S2.

### 2.2. Subjects

#### 2.2.1. Egg collection and incubation

The herring gulls used in this study are part of a larger research project and were raised and tested at the avian research facilities of Ghent University (Lab number LA1400452), located at the Wildlife Rescue Centre (WRC) in Ostend, Belgium. Eggs were collected in May and June 2024, from nests of roof-breeding parents, by the Research Institute for Nature and Forest (INBO) under the license of the Agentschap voor Natuur en Bos (ANB) and the ‘gull patrol’ team, authorized to remove eggs along the Belgian coasts for nuisance prevention. Collected before the pipping stage, the eggs were transported to the WRC under stable conditions for further incubation, using Brinsea Ova-Easy incubators (temperature = 37.5°C; humidity = 45%). Upon arrival, eggs were marked with a unique nest identifier and the two largest eggs, which are typically the first laid eggs of a clutch [48], were incubated. They were checked twice daily for small cracks, indicating pipping. Eggs showing signs of pipping were moved to an MS700U Hatchery (temperature = 37.2°C; humidity = 50%).

#### 2.2.2. Chick rearing

Once hatched and fully dried, the chicks received a unique combination of colour rings for identification. Feather samples were collected for sex determination via polymerase chain reaction (PCR), following the protocol outlined by [49]. This method targeted the CHD1W and CHD1Z introns using 2550F/2718R primers, with PCR conditions set to 30 cycles at an annealing temperature of 56°C. The chicks were then housed in groups of 10 in boxes with netting bottoms (size = 120 × 60 × 60 cm, LWH) within heated rooms (ambient temperature = 15–25°C; humidity = 40–80%; under natural light conditions). Each box contained a heating plate (30 × 30 cm). The semi-precocial chicks were hand-fed small pieces of fish and dog pellets soaked in water, supplemented with Akwavit, a complementary feed specially developed for fish-eating animals (Kasper Faunafood, The Netherlands). Food was available *ad libitum*. Once the chicks were at least 5 days old and their weight exceeded 60 grams, they were moved to outside enclosures (size = 500 × 205 × 265 cm, LWH), housed in stable groups of 8−10 individuals. Outside, heating plates were provided during the first few days if night-time temperatures were forecasted to drop below 5°C or in the event of adverse weather conditions such as heavy rain or storms. Food consisted of a mixture of dog pellets soaked in water and fish, provided four times per day, following the default policy at the WRC. Water was provided *ad libitum*. Individuals were tested when they were approximately 30 days old, shortly before they reached fledging age. After testing, the birds were moved to a large flight cage (approx. 180 m${,}^{2}$) for dehabituation from handling. Once they were 8−10 weeks old, birds were released in the wild, and a subset (*n* = 23) received a GPS-device.

#### 2.2.3. Behavioural test: novel object task

**Task Design:** For testing purposes, each home enclosure containing 8−10 birds was pseudo-randomly divided into two stable testing groups of four to five individuals that were familiar with each other. Within these subgroups, we ensured that nestmates were not placed in the same testing group. This arrangement allowed us to maintain consistent housing conditions when not testing, while ensuring that testing sessions consistently involved the same subgroups of four to five individuals.

**Figure 2.**
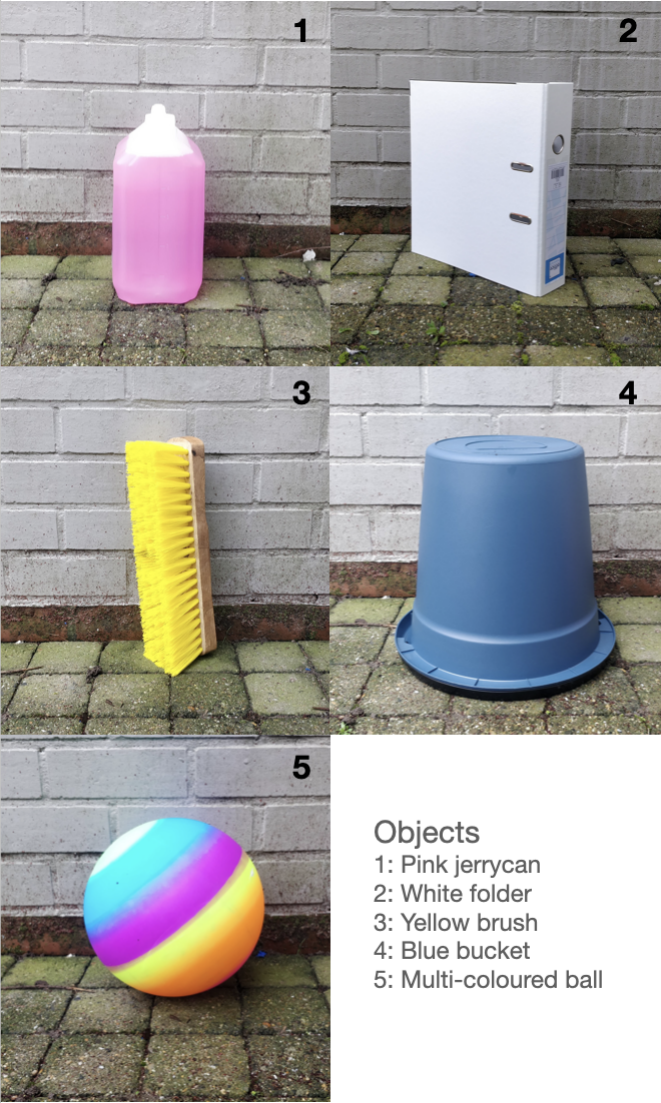
Novel or control objects.

In the ‘novel object’ condition, birds were exposed to a pseudo-randomly selected novel object. Conversely, in the ‘control object’ condition, birds were exposed to a familiar object. By placing a familiar object behind the food plate in the control condition, we ensured that responses in the 'novel object’ condition were elicited by the novelty of the object and not just the presence of the object itself (see, e.g. [2], for justification). The familiar object remained in place throughout the testing and habituation period to avoid dishabituation from the familiar object. It was replaced by the novel object only during the novel object testing sessions. To preserve the integrity of the experimental design, the novel object introduced in each of the four sessions was unique, thus, each bird’s interaction with it marked their first encounter. The experimental timeline spanned from late June to mid-July and lasted eight consecutive days. We used five objects (figure 2) of similar size (approx. the same size as a four-week-old gull), but of different colour, form and texture.

**Figure 3.**
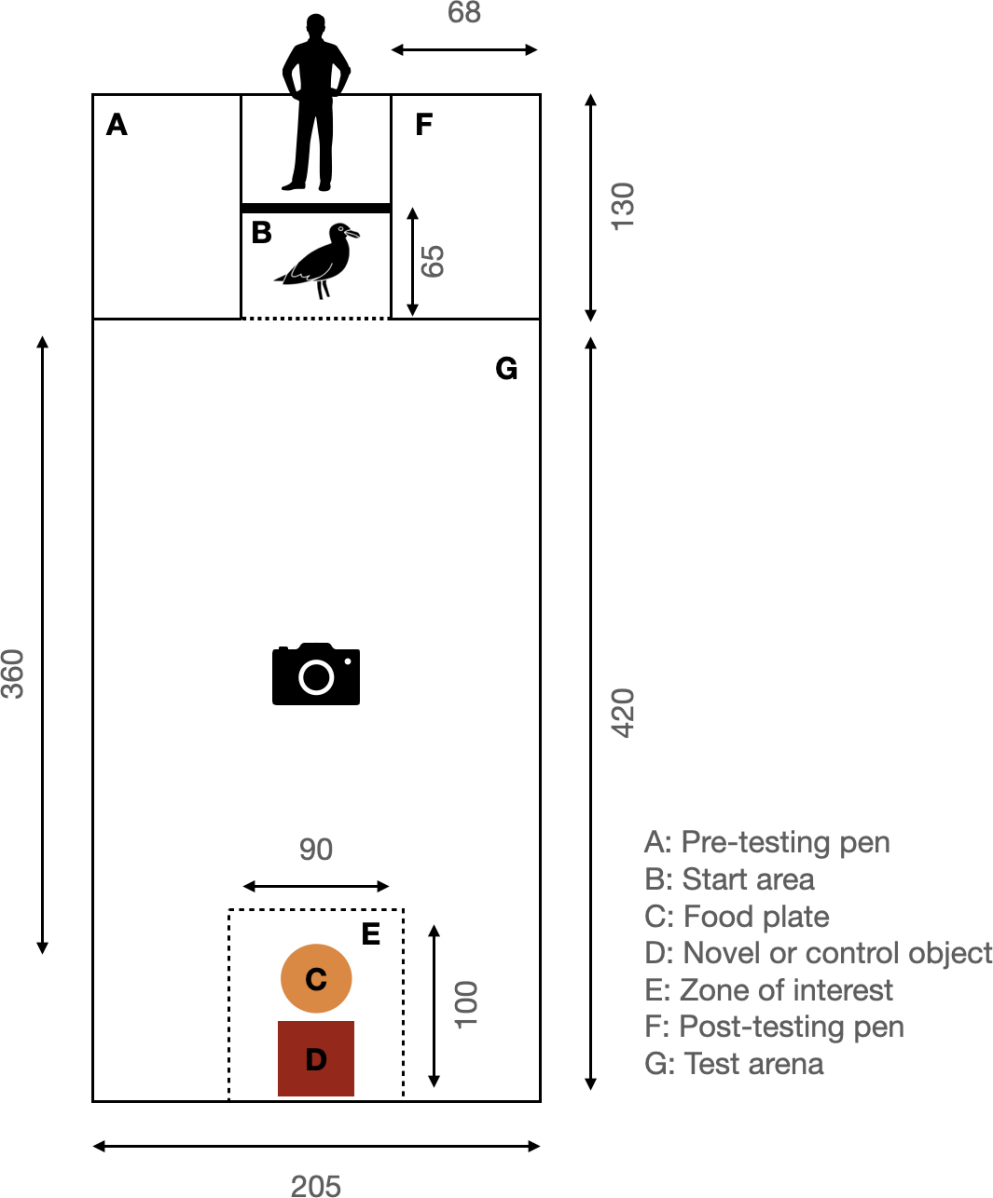
Test setup in home enclosure.

**Prior to the Task:** In preparation for the novel object task, and following a series of cognitive tests as part of another study (three tests in total), the test setup (figure 3) was introduced into the birds’ home enclosure when the birds were not present. This setup included the pre- and post-testing pens, the start area, and one of our five pseudo-randomly selected objects, which later acted as the control object in the neophobia assessments. After having introduced the test setup, birds were allowed to be accustomed to the presence of the test apparatus for a period of six days. This habituation period minimized any potential stress towards a new environment, which may influence the behavioural outcome of the test trials.

In order to distinguish the birds when they were being tested in a group, each individual was given a unique marking (marker pen, Raidex) a few days before the test, which could be easily detected by a roof-mounted camera, as colour rings were not visible in the video recordings.

**Testing Protocol:** The testing commenced after the six-day habituation period. The order of conditions was counterbalanced to incorporate control and novel object conditions, as well as individual versus group settings, with the entire sequence being repeated twice. The animals were food deprived since their last feeding moment the evening before each test at 5.30, to reduce motivational differences before testing. Testing began around 7.30 and was completed around 11.00. In both group and individual settings, individuals were given a maximum of 10 min to leave the start area and enter the test arena. Once an individual entered, the trial duration was a fixed 10 min. During this period, individuals had the opportunity to feed, but the trial continued for the full 10 min duration regardless of whether the bird first touched the food. This approach aligns with previous novel object studies [50–52]. All tests were recorded with roof-mounted cameras.

Prior to testing, all the birds were moved to the pre-testing holding pen. Next, a food plate (27 cm in diameter), completely filled with fish, and an object (novel or control, depending on the condition) were placed at the back of the enclosure, with the food plate placed in front of the object to rule out directional preference. A single bird, or group of birds, depending on the social context, was placed in the start area. The tester lifted the door of the start area after 15 s and left, giving the bird(s) access to their home enclosure (figure 3). The first 10 min period started when the door began to move, the second 10 min period started for each bird individually when it left the start area. The test session ended 10 min after the bird had left the start area in individual trials or after all birds had left the start area in group trials. Next, the tester moved the tested bird(s) to the post-testing holding pen and started a new test with a new (group of) bird(s).

#### 2.2.4. Data processing and analysis

**Video coding**: We coded all videos using the free, open-source software BORIS (Behavioural Observation Research Interactive Software) [53]. Four events were coded, namely ‘start of trial’, ‘test arena entry’, ‘eating’ and ‘zone of interest’ (see table 1 for full descriptions). Based on the coded events, we determined latencies and cumulative times. By extracting the time difference between ‘start of trial’ and ‘test arena entry’, we determined the latency to leave the start area (figure 3). In order to determine the latency to approach the food, we extracted the time difference between ‘test arena entry’ and ‘eating’. Time spent in the zone of interest (i.e. in proximity to the food reward and/or novel object, see figure 3) was calculated as the cumulative time over the length of the trial. If an individual did not perform one of the target behaviours, we assigned the maximum latency, representing the full task duration (in seconds), to that behaviour. For example, the behaviour ‘test arena entry’ has a latency of 600 s if an individual did not enter the test arena. This maximum latency applies only to latency measures; for time spent in the zone of interest (ZOI), a value of 0 was recorded if a bird did not enter the ZOI. For the group tests, we followed each bird individually to code their behaviours.

**Table 1.** Ethogram of behaviours that were coded in BORIS. The ‘zone of interest’ was defined as a fixed rectangle that included the object and the food bowl. To ensure comprehensive observation coverage, this area was expanded by the approximate body length of a 4-week-old gull (30 cm). This ensured that all relevant activities within and around the novel object were captured.

action	definition
start of trial (point event)	moment the door starts moving.
test arena entry (point event)	when the entire bird is outside the start area.
eating (point event)	when the beak touches the food.
zone of interest (state event)	the bird is considered to enter or leave the zone of interest when the front half of its body crosses the (notional) boundary of the zone.

Video coding was conducted collaboratively by multiple experimenters, with 20% of all videos being double-coded by a third experimenter to assess inter-rater-reliability (IRR) using Cohen’s Kappa. This third coder was blinded to the original coding decisions and the type of the objects (control or novel), although they were not blind to the overall study aims. Our analysis resulted in a Cohen’s Kappa of 0.89, which indicates strong agreement between coders [54].

### 2.3. Statistical analysis

Statistical analyses were conducted using R v. 4.4.1 [55]. All package version numbers are documented and managed using the renv package [56]. Mixed-effects models (LMMs) were fitted using the lme4 package [57], and parameter estimation along with *p*-values was calculated using the lmerTest package [58], via Satterthwaite’s degrees of freedom method. Model assumptions, including normality and heteroscedasticity, were assessed using the performance package [59], and transformations (log or Box-Cox) were applied where necessary.

Initial diagnostic plots indicated non-normality of residuals and heteroscedasticity in the models. To address these violations, we followed a structured approach. Each dependent variable was first fitted using the raw data. When this did not meet assumptions, a log transformation was applied. This approach sufficiently improved model fit for the ZOI duration model, and the model was refitted accordingly. For the latency to enter and latency to eat models, however, the log transformation did not resolve assumption violations. In these cases, a Box-Cox transformation was implemented, with optimal lambda values determined using maximum likelihood estimation. The estimated lambda values were λ=−0.869 for latency to enter and λ=−0.828 for latency to eat. The models were refitted using the Box-Cox transformed dependent variables, leading to improved model assumptions.

The primary objective of the analysis was to determine whether neophobic responses differed between individual and group trials. LMMs were fitted to different latency measures (latency to enter, latency to eat, and ZOI duration) under appropriate transformations (log or Box-Cox). Models were selected based on the best fit and diagnostics, with Type III sum of squares used to ensure appropriate partitioning of variance for the fixed effects. Key fixed effects included *object* (control vs. novel objects) and *context* (individual vs. group). We added *trial* as a fixed effect to account for repeated testing. Additionally, sex was contrast-coded, and included as a fixed effect to account for potential differences between males and females. For two individuals with missing data, one where the PCR failed and another where the sample was lost, a value of 0 was assigned. Initially, we fitted a full random effects structure (in line with the preregistered report) accounting for variability at the *NestID*, *GroupID* and *BirdID* levels, with specific terms for individual (*indiv_dummy*) and group (*group_dummy*) conditions to capture within-subject and within-group variation. The full random effects model was:


Latency∼Object×Context+Trial+Object×Sex+(1|NestID)+(−1+Group_dummy|GroupID)(2.1)+(−1+Indiv_dummy+Group_dummy|BirdID)

However, the full random effects structure, outlined in the preregistered report, led to over-parameterization. Consequently, non-significant interactions were dropped to simplify the model. In addition, the final models were simplified by including only random intercepts for *BirdID*, while retaining the dummy variables where the model allowed it. This approach effectively captured individual-level variability in both individual and group conditions, avoiding over-fitting. The final model structure for each latency measure is as follows:


Box-Cox(Latency to enter)∼Object+Context+Trial+Sex(2.2)+(−1+Indiv_dummy+Group_dummy|BirdID)

Box-Cox(Latency to eat)∼Object×Context+Trial+Sex(2.3)+(1|BirdID)

Log(ZOI duration)∼Object×Context+Trial+Object×Sex(2.4)+(−1+Indiv_dummy+Group_dummy|BirdID)

For models fitted on Box-Cox transformed latency data, transformation parameters were estimated using the MASS package [60]. Marginal means for the fixed effects (context and object) were computed and back-transformed to the original scale (seconds) using the Box-Cox inverse transformation, or an inverse log transformation for eating latency, with the emmeans package [61]. Random effect variances for individual (*indiv_dummy*) and group (*group_dummy*) contexts were extracted from the model outputs using the lme4 package [57]. To aid interpretation, we back-transformed the random effects by simulating random effects for 1000 individuals under both conditions (individual and group), while accounting for the covariance between *indiv_dummy* and *group_dummy* using the mvtnorm package [62]. These simulated random effects were then combined with the predicted fixed effects for each condition (individual vs. group, control vs. novel object) and back-transformed to the original scale. The inverse Box-Cox transformation was applied to latency to enter and ZOI duration, while the inverse log transformation was applied to latency to eat.

We also fitted a multivariate model on the combined dataset for *latency to enter* and *latency to eat*, using a contrast for behaviour type (*eat_vs_leave_contrast*) to account for potential correlations between the two outcomes. The multivariate model confirmed the findings from the univariate analyses, indicating consistent effects across both latency measures. However, for ease of interpretation, the results of the univariate models are presented here, as they allow a more straightforward interpretation of the individual effects of each predictor on the dependent variables. Although the multivariate model is not discussed in detail, a full walk-through of all intermediate models, including the preregistered version of the statistical analysis and the multivariate model results, is provided in the supplementary material. For exploratory purposes and to determine the robustness of our findings, we reran the analyses including all LBBG data. These analyses produced very similar results to those reported below (see electronic supplementary material, table S6).

Post hoc analyses of significant interactions were performed using estimated marginal means via the emmeans package [61], with appropriate back-transformations applied for models with transformed dependent variables. Random effect variances were compared between individual and group trials using likelihood ratio tests to assess whether separate variance components were warranted for each condition. Binary predictors were contrast-coded as (− 0.5 vs. 0.5) to optimize interpretability. Multi-collinearity concerns were minimal due to the balanced nature of the predictors, so variance inflation factor (VIF) assessments were not required. Finally, model assumptions were verified through diagnostic plots, and pairwise comparisons for significant findings were adjusted using Bonferroni–Holm corrections.

## 3. Results

Descriptive statistics for each dependent variable across the four experimental conditions are summarized in table 2. The table includes the means, standard deviations, minimum and maximum values (all in seconds) and the number of non-responses (instances where birds did not perform the target behaviour) for each condition.

**Table 2.** Descriptive statistics for each dependent variable across the four experimental conditions.

variable	condition	mean (s)	s.d. (s)	min (s)	max (s)	non-responses
latency to enter	group-control	2.34	1.41	0.87	9.40	0
	group-novel	2.58	7.52	1.08	27.92	0
	individual-control	11.30	53.14	1.16	335.56	1
	individual-novel	23.35	79.10	0.93	494.57	2
latency to eat	group-control	3.29	6.03	1.93	20.50	0
	group-novel	18.59	43.73	1.72	239.68	2
	individual-control	22.94	27.61	2.56	66.13	3
	individual-novel	146.62	118.90	2.73	367.37	24
ZOI duration	group-control	114.43	135.42	8.20	465.87	0
	group-novel	180.59	135.55	9.83	597.23	2
	individual-control	153.28	152.78	10.08	598.13	2
	individual-novel	104.16	148.34	2.27	595.44	16

All results are reported using both the transformed values (Box-Cox or logarithmic) and back-transformed values (Mean [M], s.e., CI) to facilitate interpretation on the original scale. Box-Cox transformations were applied to address non-normality and heteroscedasticity for *latency to enter* and *latency to eat*, while a logarithmic transformation was used for *ZOI duration*. In the main text, we report back-transformed values for ease of interpretation, and *p*-values for the statistical tests. The complete output of the mixed-effects models can be found in table 3.

**Table 3.** Results of linear mixed-effects models for all dependent variables, including variance components and t -values (with degrees of freedom) where applicable. Significant effects are highlighted in bold.

effect	latency to enter			latency to eat			ZOI duration		
	estimate (s.e.)	t -value (*df*)	p -value	estimate (s.e.)	t -value (*df*)	p -value	estimate (s.e.)	t -value (*df*)	p -value
*fixed effects*									
intercept	0.697 (0.021)	*t* (154.22) = 33.399		0.837 (0.015)	*t* (103.08) = 55.060		4.098 (0.126)	*t* (133.95) = 32.530	
context (group)	**−0.046** (0.022)	*t* (52.61) = −2.080	0.042	**−0.201** (0.011)	*t* (374.00) = −18.445		**0.427 (0.136**)	*t* (52.99) = 3.128	0.003
object (novel)	0.013 (0.018)	*t* (321.58) = 0.721	0.472	**0.090 (0.011**)	*t* (374.00) = 8.258		**−0.288** (0.104)	*t* (320.01) = −2.776	0.006
context × object	—	—	—	**−0.053** (0.022)	*t* (374.00) = −2.440	0.015	**1.262 (0.208**)	*t* (320.02) = 6.079	
trial	**−0.052** (0.004)	*t* (344.37) = −13.030		−0.0012 (0.0024)	*t* (374.00) = −0.507	0.613	**0.068 (0.023**)	*t* (339.53) = 2.984	0.003
sex	**−0.087** (0.029)	*t* (52.08) = −2.970	0.004	**−0.055** (0.026)	*t* (52.00) = −2.109	0.040	−0.034 (0.183)	*t* (52.06) = −0.187	0.852
object × sex	—	—	—	—	—	—	**0.550 (0.212**)	*t* (320.08) = 2.593	0.010

*variance components*									
variance (individual)	**0.018 (0.133**)	—	—	**0.007 (0.085**)	—	—	**0.748 (0.865**)	—	—
variance (group)	**0.006 (0.075**)	—	—	—	—	—	**0.192 (0.438**)	—	—
residual	0.036 (0.189)	—	—	0.013 (0.113)	—	—	1.163 (1.078)	—	—
likelihood ratio test	$\chi^{2}(2)=11.83$	—	p=0.003	—	—	—	$\chi^{2}(2)=15.815$	—	p

### 3.1. Latency to enter

Significant effects of *context* (individual vs. group) (p=0.04) and *sex* (p<0.01) were found on the birds’ latency to enter the test arena, while *object* (novel vs. control) did not have a significant effect (p=0.47). Specifically, birds tested in the *group* context entered the arena significantly faster (back-transformed M=1.91s, s.e.=0.05, 95%CI=[1.81,2.02]) than those tested *individually* (back-transformed M=2.07s, s.e.=0.09, 95%CI=[1.91,2.26]). On average, males entered the arena more quickly than females (M=1.84s, s.e.=0.068, 95%,CI=[1.71,1.98] for males; M=2.15s, s.e.=0.089, 95%,CI=[1.99,2.34] for females).

Variance analysis revealed greater individual variability in latency to enter the test zone when birds were tested *individually* (back-transformed $\sigma^{2}=2.72\;{\text{s}}^{2}$, s.d. = 1.65s) compared to when they were tested in a *group* (back-transformed $\sigma^{2}=1.22\;{\text{s}}^{2}$, s.d. = 1.10s). A likelihood ratio test confirmed that this reduction in variance in the group context was statistically significant ($\chi^{2}(2)=11.8$, p=0.02). These findings suggest that birds’ behaviour was more consistent when tested in groups.

Moreover, the estimated correlation between the individual and group random effects was high (Corr = 0.82), indicating strong repeatability of behaviour across both contexts. Birds that entered quickly when tested alone also tended to enter quickly when tested in groups.

### 3.2. Latency to eat

**Figure 4.**
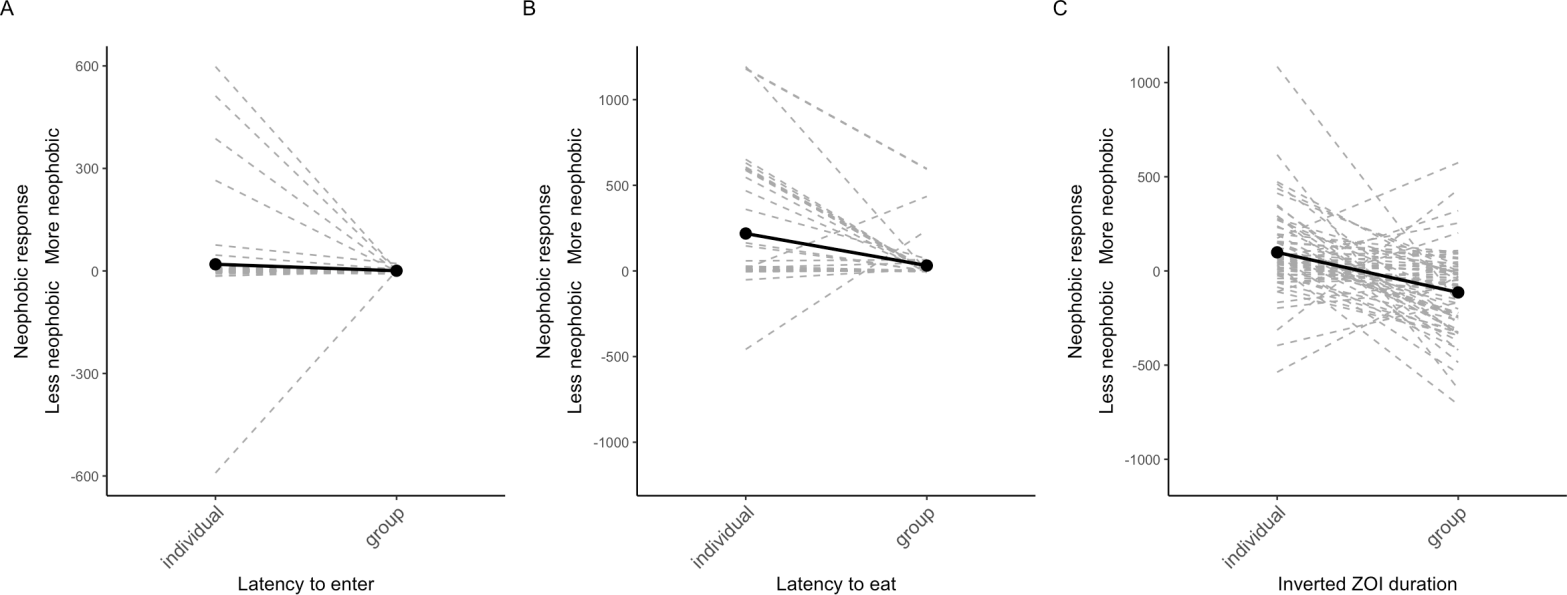
Difference plots of raw values illustrating changes in neophobic response from *individual* to *group* contexts for each dependent variable (i.e. latency to enter, latency to eat, ZOI duration). Black lines show the average response whereas dotted lines show the individual responses to illustrate the variance. In particular, plot A (latency to enter) shows a reduced variance but not a reduced average in neophobic responses across social contexts. Note that individual variation is partly masked, as lines are plotted on top of each other. Plot B (latency to eat) illustrates a reduced average neophobic response across social contexts, whereas we could not test for a reduction of variance. Plot C (ZOI duration) indicates a reduced average and a reduced variance in neophobic responses across social contexts.

Latency to eat was significantly influenced by *context* (p<0.001) and object (p<0.001). We also found a significant interaction between *context* and *object* (p=0.02). As shown in figure 4, the effect of the novel object on latency to eat was more pronounced when birds were tested individually (table 3). Specifically, birds in the *group-control* condition ate the fastest (back-transformed M=2.96s, s.e.=0.12, 95%CI=[2.74,3.21]), followed by those in the *group-novel* condition (M=3.52s, s.e.=0.17, 95%CI=[3.22,3.88]) and birds in the *individual-control* condition (M=5.20s, s.e.=0.35, 95%CI=[4.59,5.98]); birds in the *individual-novel* condition showed the longest latency to eat (M=9.81s, s.e.=1.13, 95%CI=[7.97,12.64]). Notably, in the *individual-novel* condition, 24 birds did not eat at all during the trial (table 2).

In addition, we found a main effect of *sex* (p<0.04), as males (M=4.00s, s.e.=0.25, 95%,CI=[3.56,4.56]) were, on average, faster to eat than females (M=4.88s, s.e.=0.35, 95%,CI=[4.27,5.68]).

Variance analysis indicated that the full model, which retained the random effect structure for both individual and group conditions, did not provide a better fit than the reduced model (likelihood ratio test: $\chi^{2}(2)=1.04$, p=0.59), indicating no significant difference in variance between the two conditions.

### 3.3. Zone of interest (ZOI) duration

Analysis of the time spent in the ZOI indicated significant effects of *context* (p=0.003) and *object* (p=0.005) and a significant interaction between them (p<0.001) (table 3). Birds in the *group-novel* condition spent the most time in the ZOI (back-transformed M=111.8s, s.e.=13.39, 95%CI=[88.3,141.7]), followed by those in the *individual-control* condition (back-transformed M=98.4s, s.e.=15.44, 95%CI=[72.0,134.4]). Birds in the *group-control* condition spent slightly less time in the ZOI (back-transformed M=80.2s, s.e.=9.60, 95%CI=[63.3,101.6]), while those in the *individual-novel* condition spent the least time in the ZOI (back-transformed M=38.8s, s.e.=6.09, 95%CI=[28.4,53.1]). Notably, in the *individual-novel* condition, 16 birds did not enter the ZOI at all (table 3).

We also observed a significant interaction between *object* × *sex* (p=0.010), indicating that males and females responded differently to novel vs. control objects. Females spent more time in the ZOI in the *control* condition (back-transformed M=103.1s, s.e.=15.56, 95%CI=[76.4,139.1]), compared to the *novel* condition (back-transformed M=58.7s, s.e.=8.85, 95%CI=[43.5,79.2]). In contrast, males showed a more stable response across object conditions, spending a similar amount of time in the ZOI for both control (back-transformed M=75.7s, s.e.=11.84, 95%CI=[55.5,103.2]) and novel objects (back-transformed M=74.7s, s.e.=11.67, 95%CI=[54.7,101.8]). This suggests that females exhibited a stronger response to object novelty than males.

Variance analysis indicated greater individual variability in ZOI duration when birds were tested *individually* (back-transformed $\sigma^{2}=15380.41\;{\text{s}}^{2}$, s.d. = 124.02s) compared to when they were tested in a *group* (back-transformed $\sigma^{2}=10220.50\;{\text{s}}^{2}$, s.d. = 101.10s). A likelihood ratio test confirmed that this reduction in variance in the group context was statistically significant ($\chi^{2}(2)=15.815$, p<0.001), suggesting that birds’ behaviour was more consistent when tested in groups.

A high estimated correlation between the individual and group random effects (Corr = 0.68) was observed, suggesting that birds that spent more time in the ZOI when tested individually also tended to do so when tested in a group. This indicates consistent behaviour across both social contexts.

## 4. Discussion

This study investigated how social context affects neophobic responses in juvenile herring gulls, specifically whether the presence of conspecifics influences both average behaviour and behavioural variability. We tested three hypotheses: (i) the risk dilution hypothesis, which posits that individuals share the perceived risk among group members, predicting reduced neophobia and reduced variance in groups; (ii) the negotiation hypothesis, which suggests that individuals negotiate who will approach first, predicting increased neophobia but reduced variance; and (iii) the social conformity hypothesis, which proposes behavioural synchronization within the group, resulting in no change in average neophobic responses but reduced variance in groups.

We found that individuals tested in groups were, on average, quicker to enter the test arena and eat than when they were alone. They also spent more time in the ZOI. For the latter two measures, this group effect was most pronounced when a novel object was placed behind the food (compared to a control object), suggesting reduced neophobia in a group context. In addition, we found reduced variance in group contexts for measures of entering the test arena and time in the ZOI but not for latency to eat.

Overall, these results are consistent with the risk-dilution hypothesis, whereby perceived risk is shared among individuals, resulting in each bird perceiving a lower level of threat when in group than when alone with a novel object. Observing conspecifics in the vicinity of a novel object and feeding next to it appears to reduce individual neophobic responses, as each bird likely perceives the risk to be shared by the group. This is consistent with previous studies showing that social animals often rely on the presence of the group to make quicker decisions and engage in potentially risky situations [6,17,18]. In contrast, our findings did not support either the negotiation hypothesis or the social conformity hypothesis. However, both hypotheses have been supported by other studies that have found no change or even an increase in the average neophobic response in the group context [26–32,34,35 ]. The inconsistency between studies could be due to a number of factors. In our study, for example, all individuals were of a similar age, size and had very similar early-life experiences. But in natural settings, groups are not always so homogeneous. In such settings, social conformity or negotiation mechanisms may play a more important role [63,64]. For example, more experienced individuals may assume a leading role in approaching novel objects, while others may follow their lead. This hierarchical or conformist response may result in the delayed engagement of other individuals, particularly subordinates, until the perceived risk is mitigated by the actions of more experienced group members.

More generally, the effect of social context on neophobia may be dependent on the ecological niche of the species, which may explain the discrepancy between studies. Herring gulls are considered a highly social species. Species with different ecological niches or social structures may adopt alternative social mechanisms [31]. Furthermore, in more solitary or less adaptable species, social learning will be less important overall, so neophobic responses may not vary as flexibly with social context at all. For example, a study conducted by Echeverria and Vassallo [65] examined neophobic responses in house sparrows (*Passer domesticus*). Even though birds were observed in groups, they continued to approach feeders individually, exhibiting a heightened level of neophobia. This solitary approach, even in a group setting, may reflect a social structure that prioritizes individual foraging, thereby increasing perceived risk in novel situations due to the lack of social reinforcement. Thus, the effect of social context on neophobic behaviour is likely to differ between species and ecological settings.

The general pattern of our results suggests reduced neophobia in the group context. Interestingly, birds in the group context spent even more time in the ZOI when the novel object was present than when the control object was present (resulting in ‘negative’ neophobia scores; see figure 4). This finding contrasts with the typically observed neophobic response, where novel stimuli are typically associated with reduced time spent near the object [10]. However, a similar pattern was observed in a group of shiny cowbirds (*Molothrus bonariensis*), which spent more time near the feeder when a novel object was present compared to the control condition [65]. Other studies have also found that once individuals reached the novel object, they spent more time interacting with it in a group context [26,27,31]. However, in these studies, there was no control condition, which makes the interpretation of the results difficult. One possible explanation for our results and those of Echeverría and Vassallo [65] is that the presence of conspecifics reduced fear responses sufficiently to allow individuals to approach and feed despite the novel object, but not enough to reduce them altogether, resulting in some degree of vigilance. Thus, birds in groups may have balanced their reduced fear with the need for heightened vigilance [66], leading to longer feeding times in the novel-object condition. Alternatively, being in a social context may have encouraged individuals to explore more in the novel object condition [26,67]. In the control condition, where the object was familiar, there was less need for exploration as the birds were already habituated to the object [68]. This difference in exploration could potentially also explain the increased time in the ZOI. However, at this point, our current data analysis does not permit a definitive distinction between these possible explanations.

Although the latency to eat and the time spent in the ZOI were influenced by the presence of the novel object, no such effect of the object was observed for the latency to enter. Note that we did find a main effect of social context as juvenile herring gulls tested in groups generally exited the start area more quickly than those tested individually. The absence of an effect of object on start latency could be due to our test set up. Prior to the start of the trial, the birds were unable to see the object. Given the rapidity with which they exited the start area, it is probable that they became aware of the novel object only after leaving this start area.

Additionally, we found a strong correlation between the individual and group random effect for latency to enter and ZOI duration (note that for latency to eat, we were unable to assess repeatability as our model structure did not allow for this). This correlation suggests that the average behavioural response of an individual was consistent between social contexts, for instance, in the time spent in the ZOI. Our findings align partly with those of [27], who observed consistency in juvenile raven behaviour between individual and dyadic contexts (although this effect diminished in groups of three to six individuals). Overall, this suggests that the presence of conspecifics partially reduced, but did not completely eliminate, inter-individual variation in behaviour. From a methodological point of view, this may indicate that testing individuals alone may be useful to test intrinsic abilities, information that could then be used to further explore group dynamics (e.g. leaders vs. followers).

Finally, we found sex-specific behavioural differences in our juvenile gulls. The observed differences in neophobic responses were unexpected, as herring gulls exhibit early sexual dimorphism but do not reach sexual maturity until four years of age. Nevertheless, females spent more time near the object in the control condition compared to the novel condition, whereas males showed a more consistent response across both object conditions. This could indicate that female herring gulls are more neophobic than male herring gulls. We did not observe an interaction between sex and object condition for the other two measures, although we did find a main effect as males were generally faster than females on both novel and control trials. As our study was not specifically designed to assess sex differences, and given the constraints of our sample size, we cannot rule out the possibility that a larger dataset or a different experimental design might reveal an interaction effect for latency to eat as well. Previous research on sex differences in neophobia among avian species is limited and remains inconclusive. Although some studies suggest that males are less neophobic [69], others report the opposite [70–72] or find no significant differences at all [34,73,74]. These discrepancies may arise from variations in the way neophobia is assessed, making direct comparisons challenging. The functional implications of sex differences in neophobia remain unclear but could influence broader behavioral patterns in the wild, such as sex-specific foraging strategies.

A potential limitation of our study is that we worked with juvenile, hand-reared herring gulls in a captive controlled environment. This may limit the generalizability of our findings to wild populations or adult birds. For example, a meta-analysis has shown that wild-caught birds tend to have higher baseline neophobia than captive-bred individuals, probably due to the greater environmental variation encountered by wild birds [73]. Although the lower baseline neophobia observed in captivity might reduce the likelihood of detecting group effects, our results demonstrate that such effects can still be identified. Furthermore, in other avian species, age and environmental familiarity have been shown to significantly influence neophobic responses [9,10,26]. However, previous research from our laboratory on captive herring gulls reared under similar conditions [75], pre-print, and on wild-reared chicks from a neighbouring colony [76] shows that strictly controlled testing of birds at this age provides ecologically relevant data on potential behavioural expression. In addition, prior research on wild herring gulls has shown no age-related differences in latency to approach novel objects [38], suggesting our results might extend across different age groups. Finally, all chicks in our study had very similar prior life experiences, minimizing sampling bias. This approach addresses potential challenges encountered in wild populations, where tested animals may not be fully representative of the broader population due to prior habituation to specific novelties or situations, or already developed dominance hierarchies.

To conclude, our findings demonstrate that social context plays an important role in shaping neophobic behaviour in juvenile herring gulls. For social species, group living may lower the cost of learning for individuals, as they can rely on the actions of experienced conspecifics to evaluate potential threats, reducing the need for independent assessments of novel stimuli [77]. This is under the assumption that peers provide accurate assessments of risk. Such collective risk assessment can enable more efficient exploration and engagement with the environment, mitigating the full cost of individual trial and error. Especially in rapidly changing or urbanized landscapes, where animals frequently encounter novel stimuli, the ability to draw on social cues could likely offer a distinct advantage to social species [6,64,78–81].

## Data Availability

All necessary data, scripts and code required to replicate our study’s findings will be made openly accessible at the article’s OSF repository: [82] Supplementary information, supporting our results, will also be made available at this repository. Due to the large volume of videos, totalling over 400GB, the videos will be made available upon request by contacting the senior author at frederick.verbruggen@ugent.be. Supplementary material is available online [83].
